# Supplementary material for: The Impact of Treatment for Smoking on Breast Cancer Patients’ Survival
Source: Cancers (Basel). 2022 Mar 12;14(6):1464. doi: 10.3390/cancers14061464 (PMC8946483; doi:10.3390/cancers14061464)
Supplement: Supplementary file 1 [file cancers-14-01464-s001.zip › cancers-1604789-supplementary.pdf]

cancers

Article

The Impact of Treatment for Smoking on Breast Cancer Patients' Survival

Akshara Singareeka Raghavendra [1], George Kypriotakis [2], Maher Karam-Hage [2,3], Seokhun Kim [2], Mazen Jizzini [4], Kareem S. Seoudy [5], Jason D. Robinson [2], Carlos H. Barcenas [1], Paul M. Cinciripini [2], Debu Tripathy [1] and Nuhad K. Ibrahim [1,*]

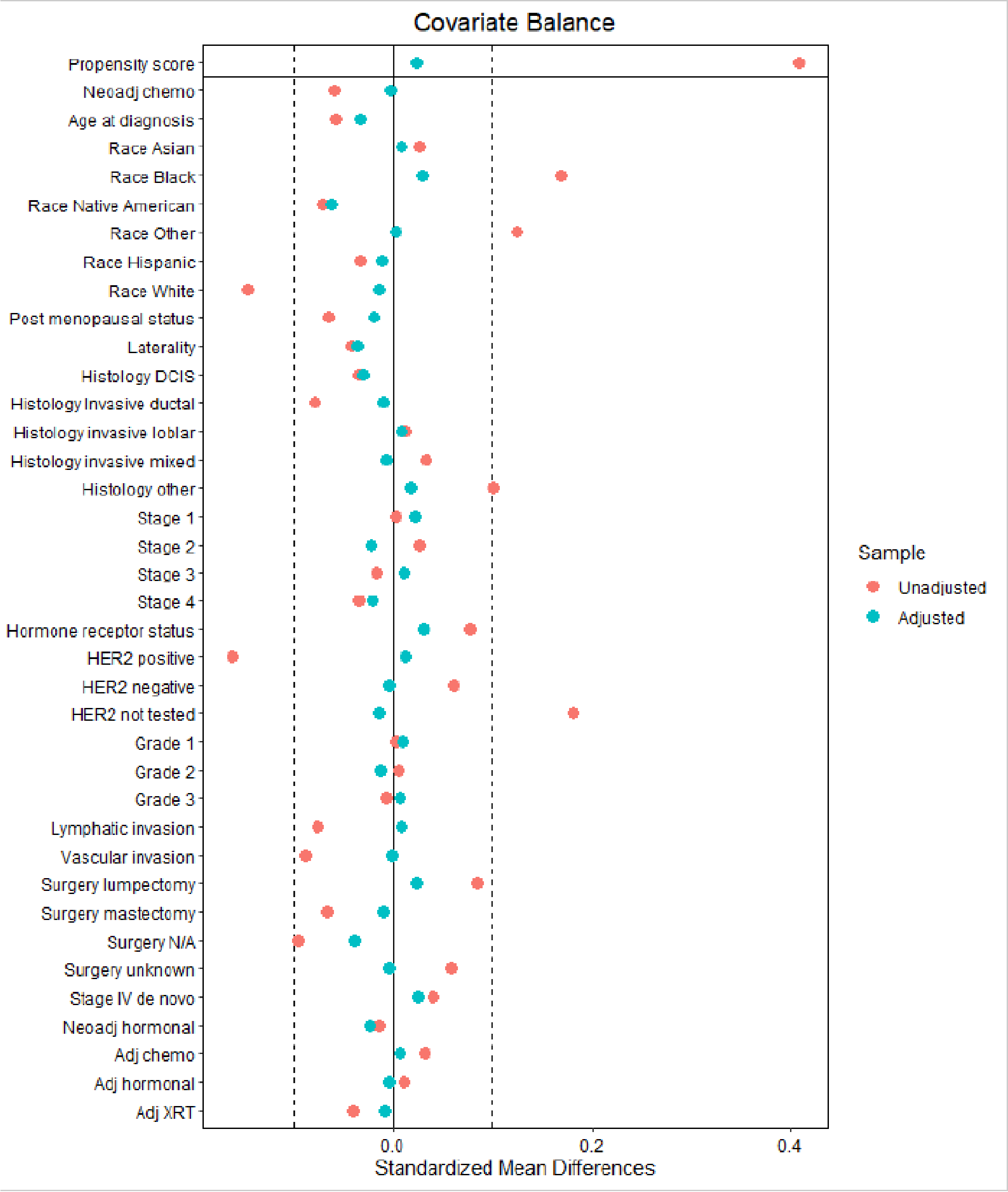

Figure S1. Covariate balance – Standardized Mean Differences.

Table S1. Covariate balance between 2 groups.

Diff. Un = Unadjusted standardized mean difference before weighting			
Diff. Adj = Adjusted standardized mean difference after weighting			
**Balance Measures**	**Diff. Un**	**Diff. Adj**	**M. Threshold**
Age at diagnosis	-0.0588	-0.0338	Balanced, <0.1
Race Asian	0.0256	0.008	Balanced, <0.1
Race Black	0.1684	0.029	Balanced, <0.1
Race native American	-0.0704	-0.0622	Balanced, <0.1
Race Other	0.1242	0.0028	Balanced, <0.1
Race Hispanic	-0.0335	-0.0106	Balanced, <0.1
Race White	-0.147	-0.0141	Balanced, <0.1
Post-menopausal status	-0.0646	-0.0201	Balanced, <0.1
Laterality	-0.0417	-0.0356	Balanced, <0.1
Histology - DCIS	-0.0352	-0.0311	Balanced, <0.1
Histology - Invasive ductal	-0.0795	-0.0105	Balanced, <0.1
Histology - Invasive lobular	0.0127	0.0083	Balanced, <0.1
Histology - Invasive mixed ductal/lobular	0.0331	-0.0064	Balanced, <0.1
Histology - Other	0.1008	0.0176	Balanced, <0.1
Overall stage 1	0.0024	0.0226	Balanced, <0.1
Overall stage 2	0.0265	-0.0219	Balanced, <0.1
Overall stage 3	-0.0161	0.0113	Balanced, <0.1
Overall stage 4	-0.0341	-0.0206	Balanced, <0.1
Hormone receptor status	0.0774	0.0303	Balanced, <0.1

HER2 Positive	-0.162	0.0125	Balanced, <0.1
HER2 Negative	0.0606	-0.0047	Balanced, <0.1
Not tested	0.1805	-0.0138	Balanced, <0.1
Grade 1	0.0021	0.0091	Balanced, <0.1
Grade 2	0.0057	-0.0125	Balanced, <0.1
Grade 3	-0.007	0.0071	Balanced, <0.1
Lymphatic invasion	-0.0757	0.0075	Balanced, <0.1
Vascular invasion	-0.088	-0.002	Balanced, <0.1
Surgery- Lumpectomy	0.0841	0.0236	Balanced, <0.1
Surgery- Mastectomy	-0.067	-0.0097	Balanced, <0.1
Surgery- N/A(stage IV/not done)	-0.0951	-0.0393	Balanced, <0.1
Surgery- Unknown	0.0574	-0.0046	Balanced, <0.1
Stage IV denovo	0.0395	0.0253	Balanced, <0.1
Neoadjuvant chemotherapy	-0.0603	-0.0027	Balanced, <0.1
Neoadjuvant hormonal	-0.0135	-0.024	Balanced, <0.1
Adjuvant chemotherapy	0.0322	0.0062	Balanced, <0.1
Adjuvant hormonal	0.0103	-0.0047	Balanced, <0.1
Adjuvant radiation (XRT)	-0.0403	-0.0089	Balanced, <0.1
Balance tally for mean differences			
	count		
Balanced, <0.1	38		
Not Balanced, >0.1	0		
Variable with the greatest mean difference			
Variable Diff. Adj M. Threshold			
Race - Native American -0.0622 Balanced, <0.1			

Table S2. Demographic and disease characteristics of study subjects by survival status.

Characteristic	Alive with no evidence of disease No. (%) (n = 1503)	Dead with disease No. (%) (n = 326)	*P*
Abstinence status			0.0768
Yes	179 (11.91)	34 (10.43)	
No	1,324 (88.09)	292 (89.57)	
Race			0.0181
White	1,123 (74.72)	237 (72.70)	
Black	176 (11.71)	54 (16.56)	
Hispanic	163 (10.84)	25 (7.67)	
Asian	24 (1.60)	5 (1.53)	
Native American	2 (0.13)	2 (0.61)	
Other	15 (1.00)	3 (0.92)	
TP participation			0.048
Yes	474 (31.54)	106 (32.52)	
No	1029 (68.46)	220 (67.48)	
Stage			< 0.001
I	562 (37.39)	29 (8.90)	
II	640 (42.58)	95 (29.14)	
III	285 (18.96)	120 (36.81)	
IV	16 (1.06)	82 (25.15)	
Hormone receptor status			0.010
Positive	1,190 (79.17)	221 (67.79)	
Negative	313 (20.83)	105 (32.21)	
HER2			0.0069
Positive	250 (16.63)	47 (14.42)	
Negative	1,223 (81.37)	269 (82.52)	
Not tested	30 (2.00)	10 (3.07)	
Menopausal status at diagnosis			0.1590
Pre	619 (41.18)	153 (46.93)	
Post	884 (58.82)	173 (53.07)	
Neoadjuvant chemotherapy			< 0.001
Yes	520 (34.60)	129 (39.57)	
No	983 (65.40)	197 (60.43)	
Neoadjuvant hormonal			0.9204
Yes	42 (2.79)	6 (1.84)	
No	1,461 (97.21)	320 (98.16)	
Adjuvant chemotherapy			< 0.001
Yes	560 (37.26)	92 (28.22)	
No	943 (62.74)	234 (71.78)	
Adjuvant hormonal			< 0.001
Yes	1,068 (71.06)	117 (35.89)	
No	435 (28.94)	209 (64.11)	
Adjuvant Radiation (XRT)			< 0.001
Yes	981 (65.27)	156 (47.85)	

No	522 (34.73)	170 (52.15)	
Grade			< 0.001
I	163 (10.84)	24 (7.36)	
II	718 (47.77)	128 (39.26)	
III	622 (41.38)	174 (53.37)	
Laterality			0.8978
Right	722 (48.04)	155 (47.55)	
Left	781 (51.96)	171 (52.45)	
Histology			0.0095
Invasive ductal	1,272 (84.63)	271 (83.13)	
Invasive lobular	116 (7.72)	33 (10.12)	
Invasive mixed ductal/lobular	65 (4.32)	10 (3.07)	
Other	50 (3.33)	12 (3.68)	
Lymphatic invasion			< 0.001
Positive	275 (18.30)	104 (31.90)	
Negative	1,228 (81.70)	222 (68.10)	
Vascular invasion			< 0.001
Positive	270(17.96)	101 (30.98)	
Negative	1,233 (82.04)	225 (69.02)	
Surgery type			< 0.001
Lumpectomy	686 (45.64)	79 (24.23)	
Mastectomy	805 (53.56)	177 (54.29)	
N/A (stage IV/not done)	6 (0.40)	53 (16.26)	
Unknown	6 (0.40)	17 (5.21)	
Stage IV de novo			< 0.001
Yes	17 (1.13)	82 (25.15)	
No	1,486 (98.87)	244 (74.85)	
Age at diagnosis, y, mean (SD)	51.34 (10.74)	49.67 (10.94)	0.419

Abbreviations: DCIS, ductal carcinoma in situ; LCIS, lobular carcinoma in situ; TP, Tobacco Treatment Program; XRT, radiotherapy.
